# Replacing Solid Snacks with Almonds or Adding Almonds to the Diet Improves Diet Quality and Compliance with the 2020–25 Dietary Guidelines for Americans: Modeling Analyses of NHANES 2017–23 Data

**DOI:** 10.3390/nu18010087

**Published:** 2025-12-26

**Authors:** Mattieu Maillot, Romane Poinsot, Maha Tahiri, Adam Drewnowski

**Affiliations:** 1 MS-Nutrition, 27 bld Jean Moulin, Faculté de Médecine la Timone, Laboratoire C2VN, 13385 Marseille Cedex 5, France; matthieu.maillot@ms-nutrition.com (M.M.); romane.poinsot@ms-nutrition.com (R.P.); 2 Nutrition Sustainability Strategies LLC, St. Petersburg, FL 33702, USA; info@nutritionsustainability.com; 3 Center for Public Health Nutrition, University of Washington, Box 353410, Seattle, WA 98195, USA

**Keywords:** almonds, diet modeling, diet quality metrics, Healthy Eating Index 2020, Nutrient Rich Food Index (NRF), snacks

## Abstract

**Background**: Healthier between-meal snacks can improve diet quality in the US and globally. **Objectives**: To assess the impact on diet quality of replacing solid snacks with almonds or adding almonds (30 g) to the diet. **Methods**: Dietary data for 4333 children (4–19 y) and 10,925 adults (>19 y) came from the National Health and Nutrition Examination Survey (NHANES 2017–23). Nutrient composition data came from the Food and Nutrient Databases for Dietary Studies (FNDDS). Substitution Model 1 replaced all solid snacks with almonds on a per-calorie basis. Model 2 exempted “healthy” snacks. Model 3 added almonds to the observed diet. The Healthy Eating Index (HEI-2020), Nutrient Rich Food Index (NRF), Mean Adequacy Ratio (MAR), and Mean Excess Ratio (MER) were measures of diet quality. **Results**: Solid snacks provided 329 kcal/day (15.6% of dietary energy), of which 58 kcal came from healthy snacks (2.9%). The 4–13 y age group consumed the most energy from snacks. Diets with almonds replacing solid snacks were lower in added sugar, sodium, and saturated (solid) fat but higher in protein, fiber, mono- and polyunsaturated fats, and magnesium. MAR dietary nutrient density scores were significantly higher, and MER scores were lower. Total HEI-2020 scores rose from 52.4 (observed) to 59.6 (Model 1) and to 60.6 in Model 2_100. The addition of almonds (30 g or 50 g) increased HEI-2020 values to 59.2 and to 61.4, respectively. Several HEI-2020 sub-scores increased as well. The greatest dietary benefits were obtained for children and young adults. **Conclusions**: Replacing solid snacks with small amounts of almonds led to higher quality modeled food patterns, especially for younger age groups. The DGA 2025–30 should address the inclusion of healthy energy-dense snacks into everyday diets.

## 1. Introduction

Foods and beverages consumed between meals now account for about 23% of daily energy intakes in the US [1,2]. American eating habits are shifting toward more eating events per day and more eating occasions away from home [3]. Frequent between-meal snacks are increasingly replacing cooked sit-down meals, particularly dinner [4]. The market for snacks in the US has grown substantially since the COVID-19 confinement and is now estimated at close to USD 200 billion per year [5].

The Dietary Guidelines for Americans (DGA) recognize that snacks can help meet energy and nutrient needs, especially for children, teens, older adults, and very active people [6]. However, the DGA also specify that between-meal snacks should provide nutrients and not just excess calories [6]. Many energy-dense snacks are important contributors of refined grains, solid fats, and added sugars [7]. Among snacks generally considered to be more wholesome are whole fruit, whole grains, vegetables, yogurts, and tree nuts [8].

The present goal was to update a previously published study based on NHANES 2009–12 data that replaced commonly eaten snacks with almonds and tree nuts [9]. In that study, replacement modeling resulted in food patterns that were lower in added sugars and solid fats and had higher nutrient density overall. In the NHANES 2009–2012 database, consumption of tree nuts in the US was still relatively low compared to other countries [10]. Almonds were the most frequently consumed item [9].

Consumption of almonds has increased very considerably since then, both in the US and worldwide [11,12]. It is generally recognized that almonds, pistachios, and other tree nuts are significant sources of some key nutrients [13,14]. Tree nuts contain high levels of plant protein, along with fiber, healthy fats, vitamin E, magnesium, and other nutrients, including flavonoids. In clinical and observational studies, the lipid profile of nuts has been associated with improved cardiometabolic health [14,15,16,17,18,19].

Modeling analyses, based on the most recent NHANES data sets, can inform future editions of the DGA [8,20]. The present objective was to model the impact of replacing typical solid snacks in the US diet with equi-caloric amounts of almonds. Variables of interest were added sugars, sodium, saturated fats, and solid fats, which are nutrients of public health concern. We also examined the modeled food patterns for protein, fiber, mono- and polyunsaturated fats (MUFA and PUFA), oils, potassium, and magnesium. The Healthy Eating Index 2020 was a measure of adherence to the DGA.

A secondary objective was to identify those population subgroups whose diets could be helped the most by replacing solid snacks with almonds. We anticipated that those subgroups would include children and teenagers. In general, those populations consume the most calories from snacks, consume the least healthy snacks, and have the least nutrient-rich diets [21,22]. We also examined the impact of adding small amounts of almonds to the diet across age and sociodemographic groups.

## 2. Materials and Methods

### 2.1. The National Health and Nutrition Examination Survey

The present analyses were based on recent cycles of the National Health and Nutrition Examination Survey (NHANES) 2017–2020 and 2021–2023 [23,24]. There were 15,258 NHANES participants aged >4 y with two completed days of 24 h dietary recall. This sample included 4333 children aged 4–19 y and 10,925 adults aged >19 y. The sample was stratified by sociodemographic variables. Sex was male or female. Age groups were 4–8 y, 9–13 y, 14–19 y, 20–30 y, 31–50 y, 51–70 y, and >71 y. Ethnicity was classified as: Mexican, Non-Hispanic Black, Non-Hispanic White, Other Hispanic, and Other race. The IPR cut points were: <1, 1–1.99, 2–3.49, and >3.5.

The dietary component of NHANES is known as the What We Eat in America (WWEIA) study [24]. NHANES data collection uses a multiple-pass method conducted by trained interviewers. NHANES participants reported the type and amounts of foods and beverages consumed during the preceding 24 h. The timing and type of eating occasion (meal or snack) were recorded as well. The first 24 h recall was conducted in person, and the second 24 h recall was conducted by telephone.

### 2.2. Identification of Between-Meal Snacks

To separate meals from meal snacks, the time course of the NHANES dietary intakes was divided into 19 eating occasions, as defined by NHANES participants. Snacks, Afternoon snacks, and Sandwiches or snacks were all classified as between-meal snacks. Eating occasions described in Spanish as Merienda, Entre Comida, Bocadillo, and Tentempie were also identified as snacks. Analyses of all eating occasions in the NHANES 2017–23 identified 17,164 snacks, both solid and liquid. For comparison purposes, there were 19,146 breakfasts, 19,521 lunches, and 21,057 dinners.

The present analyses considered solid snacks only (cakes, cookies, brownies, chips, crackers, candy). Beverages consumed between meals (milk, juices, soft drinks, sodas, alcohol) and beverage additions (sugar added to coffee) were not considered. Snacks were then separated into healthy snacks and less healthy snacks. Following past procedures [9], healthy snacks were defined as whole fruits, non-starchy vegetables, salads, whole grain foods (with >50% whole grains by weight), nuts and seeds, soy products, pulses, and unsweetened plain yogurts. Healthy snacks were defined by WWEIA codes beginning with 6402, excluding 6416 and 6418 [25].

### 2.3. The Food and Nutrient Database for Dietary Studies FNDDS

The Food and Nutrient Database for Dietary Studies (FNDDS) [25] was used to calculate energy and nutrient intakes of NHANES participants. Nutrient composition data from FNDDS 2017–2019, FNDDS 2019–2020, and FNDDS 2021–2023 were used because food identification codes were the same as in NHANES 2017–2023. When food ID codes differed between FNDDS periods, the most recent food code was used.

### 2.4. The Food Patterns Equivalents Database FPED

Foods in the FNDDS database were matched to the USDA Food Patterns Equivalents Database (FPED) using food identification codes [26]. The USDA FPED translates the nutrient content of 100 g of each food into oz equivalent or cup eq. of food groups featured in the DGA. Since FPED was only available for the NHANES cycle of 2017–2020, 29 foods or beverages consumed exclusively during the 2021–2023 NHANES cycle were matched to existing foods in the FPED file.

### 2.5. Composite Nutrient Profile of the Almond Snack

**Figure 1:**
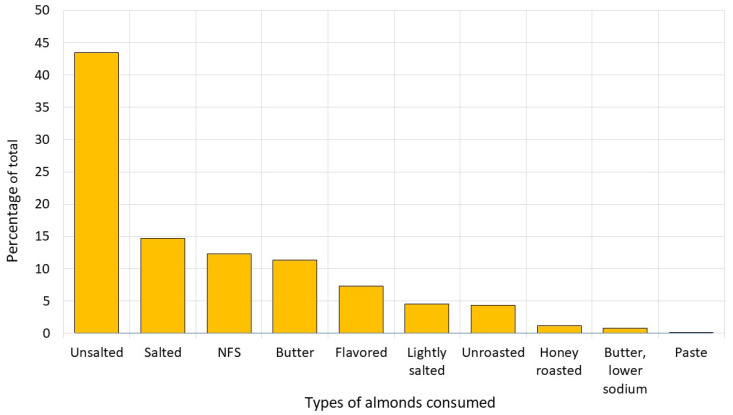
Types of almonds consumed by NHANES participants used to calculate energy and nutrient content of the composite almond snack.

NHANES 2017–23 participants consumed 10 different forms of almonds (see Figure 1 below). The composite nutrient profile of the almond snack was based on the nutrient composition of different types of almonds, weighted by consumption frequency. Unsalted almonds were the most frequently consumed form, with a weight of 43.5%. Energy and nutrient content of the composite almond snack per 30 g serving was as follows: energy, 182 kcal; carbohydrates, 6.16 g; fiber, 3.16 g; protein, 6.11 g; total fat, 16.0 g; saturated fat, 1.34 g; monounsaturated fat, 9.90 g; polyunsaturated fat, 4.04 g; magnesium, 81 mg; sodium, 40.2 mg; added sugar, 0.14 g; oils, 10.6 g; solid fat, 0.05 g.

### 2.6. Substitution and Addition Modeling Analyses

Three types of models were developed. In the substitution models, solid snacks were replaced with the composite almond snack on a per-calorie basis. In this way, 300 kcal of solid snacks were replaced with the quantity of almonds corresponding to 300 kcal. Model 1 replaced 100% of all solid snacks with the almond snack. Model 2 exempted healthy snacks as defined above. Model 2_50 replaced 50% of the remaining snacks with almonds; Model 2_100 replaced 100% of snacks with almonds.

Addition modeling was conducted as well. In the addition models, a fixed amount of the composite almond snack was added to the diet of all NHANES participants with the attendant increases in dietary energy. Model 3 (1 serving) added 30 g of almonds per day, equivalent to one serving, as recommended in many guidelines (1). Model 3 (2 servings) added 50 g of almonds per day, following recommendations from a recent consensus paper [12].

### 2.7. Diet Quality Metrics

Multiple diet quality metrics were developed, both food- and nutrient-based. The goal was to make sure that the modeled improvements in diet quality were not limited to any one assessment tool.

The Healthy Eating Index 2020 (HEI-2020), a measure of compliance with the Dietary Guidelines for Americans [27,28], is a numeric score that ranges from 0 to 100 points. The HEI-2020 is the sum of nine adequacy components: total vegetables, greens and beans, total fruit, whole grains, dairy, total protein, seafood and plant protein, and fatty acid ratio (monounsaturated fatty acids (MUFAs) to polyunsaturated fatty acids (PUFAs)), as well as four moderation components: sodium, refined grains, saturated fat, and added sugar. The 13-component HEI-2020 was calculated based on two 24 h dietary recalls for the observed and modeled diets for each NHANES participant.

The Nutrient Rich Foods (NRF) index or NRF9.3 [29,30] is the sum of percent daily values (DV) for nine qualifying nutrients called “NR” (proteins, fibers, calcium, iron, magnesium, potassium, vitamin C, vitamin A, and vitamin D) minus the sum of percent DV for three disqualifying nutrients called “LIM” (saturated fats, added sugars, and sodium) (3). Reference DV for the present NRF 9.3 were based on the US Food and Drug Administration (FDA) values for a 2000 kcal/day diet [8,20]. The standard reference amounts were 50 g for protein, 28 g for fiber, 900 RAE for vitamin A, 90 mg for vitamin C, 20 mcg for vitamin D, 1300 mg for calcium, 18 mg for iron, 3500 mg for potassium, and 420 mg for magnesium, and the maximum recommended values were 50 g for added sugar, 20 g for saturated fat, and 2300 mg for sodium. Each daily nutrient intake was adjusted for 2000 kcal. Percent DVs for nutrients were truncated at 100% so that an excessively high intake of one nutrient could not compensate for the dietary inadequacy of another. In LIM, only the share in excess of the recommended amount was considered.

The mean adequacy ratio (MAR) is the mean content of key nutrients in relation to their recommended daily intake [31]. The MAR calculation used 21 nutrients: proteins, fiber, vitamins B1, B2, B6, B9, B12, C, D, E, and A, as well as calcium, potassium, iron, magnesium, zinc, copper, selenium, linoleic acid (LA), and alpha-linolenic acid (ALA). Each nutrient’s ratio was capped at 100%, ensuring that a high intake of one nutrient could not compensate for the low intake of others, as indicated in Equation (1):

(1) $$MAR=\frac{1}{23}\sum_{n=1}^{23}\min\left(100,\frac{{intake}_{n}}{{reco}_{n}}\times 100\right),$$

where *intake_n_* is the total daily intake of *nutrient_n_* and *reco_n_* is the recommended value.

MAR ranged from 0 (no nutrient intake) to 100 (coverage of all 21 nutrients). The recommended values came from the Dietary Reference Intakes in DGA 2020–2025 [8] and those issued by the Institute of Medicine [32,33] (See Supplemental Table S1).

The mean excess ratio (MER) was the mean content of sodium, saturated fatty acids (SFA), and added sugar in relation to DGA 2020–25 recommended values [34]. Additionally, energy, added sugars, saturated fats, sodium, solid fats, total fats, proteins, carbohydrates, MUFAs, PUFAs, oils, fiber, and magnesium were calculated as mean intakes per day and as percentages of daily values.

### 2.8. Plan of Analysis

Substitution analyses were conducted for the total sample and by sociodemographic groups (sex, age group, IPR, race/ethnicity). Each scenario of modeled diets was compared to observed diets on multiple measures of diet quality: HEI-2020, MAR, and MER, as well as on selected nutrients for the whole sample and by sociodemographic strata. The significance was estimated using Student’s *t*-test.

Then, general linear models (GLM) were used to statistically compare the improvement of diet quality obtained either from a substitution of snacks with almonds or from the addition of almonds between sociodemographic groups. A *p*-value lower than 5% was considered significant. All analyses were weighted to account for the complex survey design of NHANES data, ensuring that the findings were robust and representative of the US population. Finally, the mean amount of all consumed snacks and non-healthy snacks, as well as the mean quantity of almonds used in Models 1 and 2, were estimated by age group. Data analyses used R software 4.4.3.

## 3. Results

### 3.1. The Contribution of Snack Energy to the Total Diet

Most NHANES participants (13,380 out of 15,258 or 88.1%) consumed at least one solid snack on the 2 days of NHANES dietary recalls. Table 1 shows that solid snacks provided 328.8 kcal/day or 15.6% of total daily energy. Men derived more calories from snacks than did women (354 kcal vs. 305 kcal), but women had a higher percent of energy from snacks (women 16.4%; men 14.7%) (*p* < 0.001). Solid snack energy was highest among children aged 4–8 y (363 kcal/day or 20.6%) and older children aged 9–13 y (390 kcal/day or 19.2%) (*p* < 0.001). Solid snack calories declined with age, with the lowest values found in the 20–30 y age group (303 kcal or 13.6%).

Only 58 kcal/day (or 2.8% of total intakes) came from healthy snacks. Children aged 9–19 y consumed only 36 kcal/day (or 1.85%) from healthy snacks. Older groups consumed less snack energy but more healthy snacks: 75 kcal/day (3.6% of total) for the 51–70 y age group and 65 kcal/day (3.35%) for the >70 y age group.

Groups with higher IPR derived more energy from snacks and from healthy snacks. Solid snack calories increased with IPR (from 304 to 344 kcal/day). Calories from healthy snacks more than doubled with IPR (36.6 to 69 kcal/day), rising from 1.9% to 3.3% of total energy (*p* < 0.001). The non-Hispanic white group derived the most calories (344 kcal/day) and the highest percent of energy (16.0%) from solid snacks.

**Figure 2:**
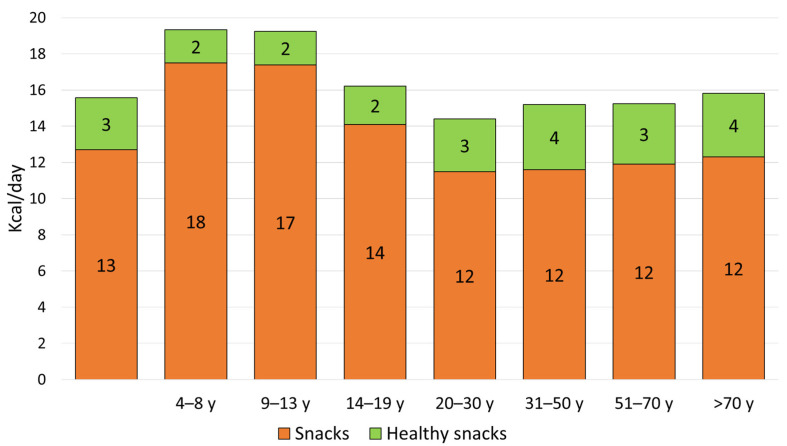
Observed percent calories from different types of snacks by age group.

The percentages calories from different types of snacks are plotted in Figure 2. Energy intakes and percent energy from snacks first increased and then declined with age. The highest values (in kcal and %kcal) were observed for children and adolescents. Adults consumed more calories overall but fewer calories from snacks. Energy from snacks classified as healthy was minimal, as were the percentages (<2%). Percent energy from healthy snacks increased with age, reaching a maximum after the age of 70.

### 3.2. Nutrient Density of Existing Solid Snacks Eligible for Replacement

Table 2 shows the nutrient content per 100 kcal of all solid snacks and the composite almond snack. Almonds were more energy-dense, with only 16 g of almonds supplying 100 kcal. Almonds had more fiber (1.7 g/100 kcal vs. 1 g/100 kcal); magnesium (44.6 mg per 100 kcal); protein (3.4 g/100 kcal vs. 2.3 g/100 kcal); and total fat (8.8 g/100 kcal), monounsaturated fat (5.5 g/100 kcal), and polyunsaturated fat (2.2 g/100 kcal). The almond snack was low in sodium (22.1 mg/100 kcal) and added sugar.

Table 3 shows the percentage of calories from WWEIA food categories that were eligible for replacement in Model 1 and Model 2. Most calories came from ice cream and frozen dairy desserts, cookies and brownies, cakes and pies, chocolate candy, tortilla, corn, and other chips, doughnuts, sweet rolls, pastries, potato chips, crackers, and popcorn. Nuts and seeds contributed 7.1% of snack calories and whole fruit another 3.7%.

### 3.3. Substitution Modeling (Models 1 and 2) Shows Higher Quality Diets

**Figure 3:**
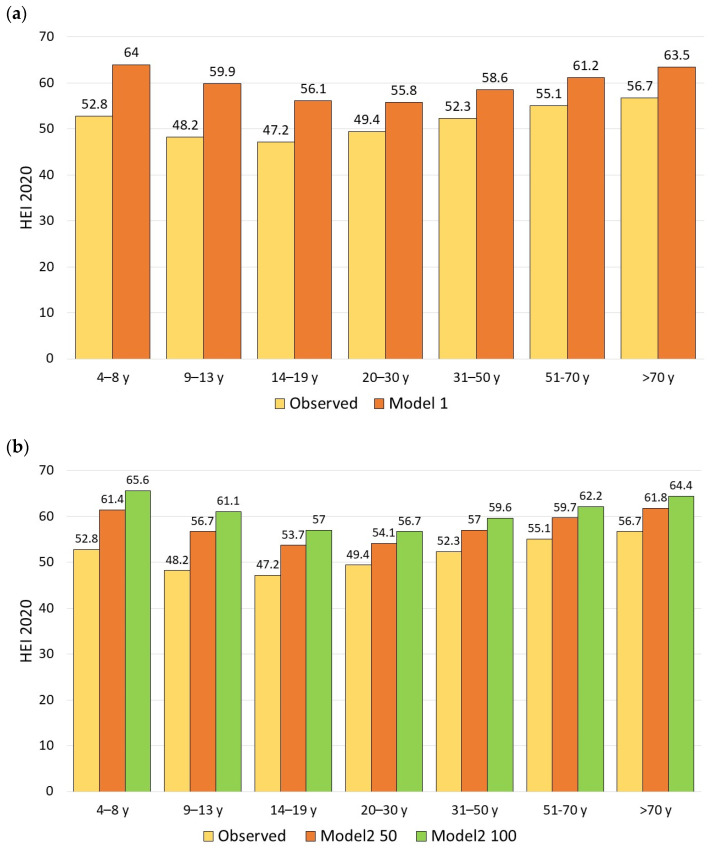
HEI-2020 values for observed diets compared to Model 1 (**a**); Model 2_50 and Model 2_100 (**b**) by age group. Model 1 replaced all snacks; Model 2 exempted healthy snacks and used 50% and 100% replacement.

Substituting solid snacks with almonds significantly increased the HEI-2020. Figure 3a shows that HEI-2020 scores rose from 52.4 to 59.6 in Model 1, an increase of 7.2 points. Figure 3b shows that HEI-2020 values in Model 2 (50%) rose from 52.4 to 57.8, an increase of 5.4 points, and those in Model 2 (100%) rose from 52.4 to 60.6, an increase of 8.2 points.

Table 4 shows that the impact of the models on HEI-2020 values varied significantly across different age groups (*p*-value of interaction < 0.001). Across all models, the most substantial improvements were seen in the 4–8 y and 9–13 y age groups. For the 9–13 y group, the HEI was equal to 48.2 in the observed diet and increased by +8.6 points (Model 2_50) to +12.8 points (Model 2_100) in the 4–8 y group and by +8.5 (Model 2_50) to +12.9 points (Model 2_100) in the 9–13 y group.

**Figure 4:**
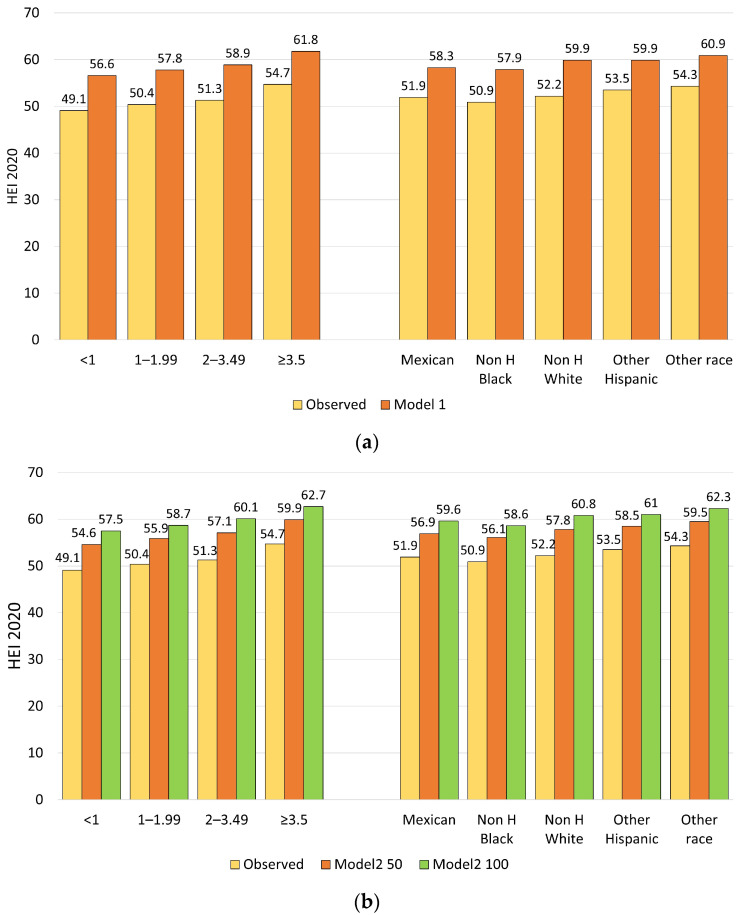
HEI-2020 values for Model 1 compared to observed diets (**a**) and HEI-2020 values for Model 2 (50% and 100% versions) compared to observed diets (**b**) by sociodemographics.

Figure 4 shows HEI-2020 values for modeled food patterns (Models 1 and 2) by sociodemographic variables: IPR and race/ethnicity.

### 3.4. Substitution Modeling (Model 1 and 2) and HEI-2020 Sub Scores

**Figure 5:**
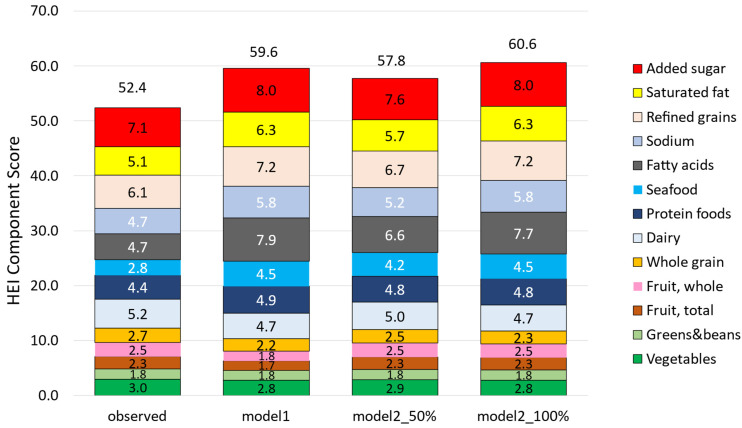
Healthy Eating Index 2020 components in observed diets and modeled food patterns (Model 1 and Models 2_50 and 2_100). Model 1 replaced all solid snacks with the composite almond snack. Model 2 exempted healthy snacks and replaced 50% or 100% of snacks with the composite almond snack. All HEI components in modeled diets were significantly different from the observed diet, except for beans/pulses, and for whole fruits and total fruits in Model 2 (50%) and Model 2 (100%).

This improvement was largely due to higher points in several categories, reflecting a significant drop in dietary added sugars and saturated fat, refined grains, and sodium. There were also increases in several adequacy components, notably the fatty acid ratio, given the higher level of polyunsaturated fats compared to saturated fats in composite almonds (Table 5). Additionally, there were increases in seafood and plant protein, along with total protein sub-scores (Figure 5). However, there were slight decreases in the dairy, whole grains, and total vegetable components, also expressed per 1000 kcal, while total fruits decreased only in Model 1.

### 3.5. Adding Almonds Improved HEI-2020 Scores and Nutrient Density of Modeled Diets

Adding almonds to the observed diet led to higher energy intakes but also to higher nutrient density scores per 1000 kcal. Table 6 shows the weight and energy content of modeled diets along with NRF9.3, MAR, and MER nutrient density scores. HEI-2020 scores increased overall (from 52.4 to 59.2 with 30 g of almonds and 61.4 with 50 g of almonds).

**Figure 6:**
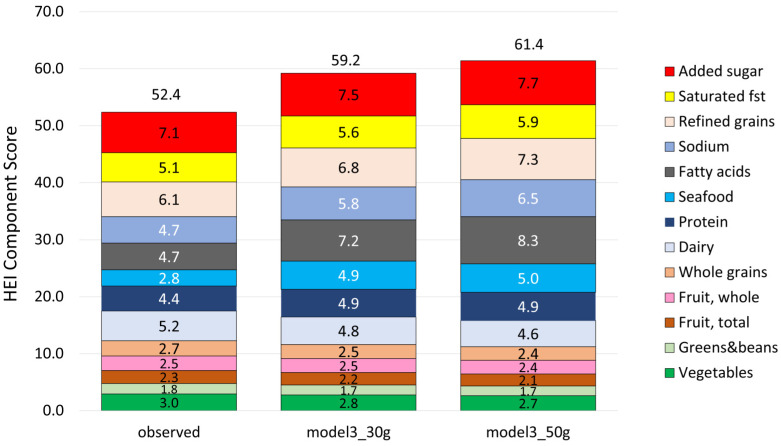
Healthy Eating Index 2020 scores and sub-scores before and after addition modeling (30 g and 50 g of almonds). All values of sub-scores in modeled diets were significantly different from the observed value.

Figure 6 shows HEI total scores and sub-scores following the addition of almonds (30 g or 50 g) to the observed diets. HEI scores (calculated per 1000 kcal) increased, and so did several sub-scores. In particular, the modeled diets were lower in refined grains, saturated fat, and sodium. The fatty acid ratios improved, and the plant protein sub-score was higher as well.

### 3.6. Greatest Benefits for Children and Adolescents in All Models

**Figure 7:**
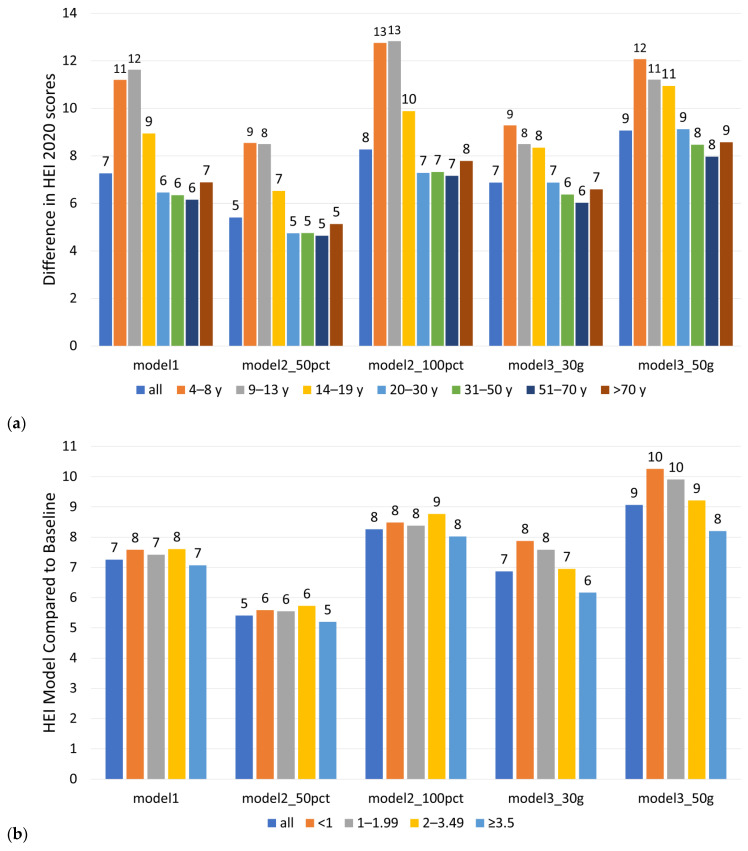

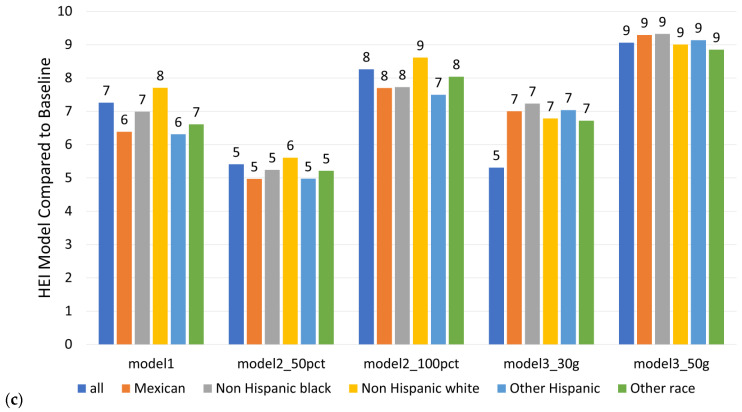
Comparison of differences between observed HEI-2020 and modeled HEI-2020 (Models 1, 2, and 3) by age group (**a**), IPT (**b**), and race/ethnicity (**c**).

Greatest improvements in dietary quality as measured by HEI were realized for children and adolescents. Those groups consume the most energy from snacks and tend to have lower-quality diets. By contrast, there were no major differences by race/ethnicity. All groups realized comparable benefits. Differences between the observed and modeled HEI by sociodemographics are shown in Figure 7. Data are shown by age group (a), IPR (b), and race/ethnicity (c).

### 3.7. Small Doses of Almonds Improve Diet Quality

**Figure 8:**
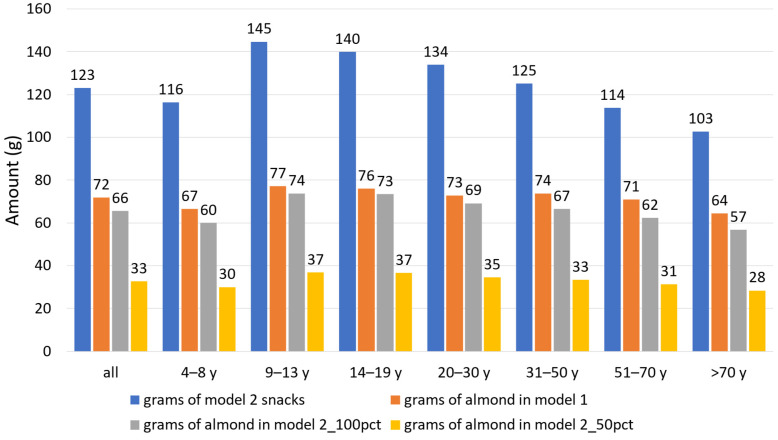
Quantity of snacks and quantity of almonds (in grams) replacing snacks in Model 1 and Model 2, total (all) and by age group. Model 1 is substitution of all solid snacks with the composite almond snack. Model 2 is substitution of 100% or 50% of all non-healthy solid snacks with the composite almond snack.

All the snacks were substituted with the composite almond on a per-calorie basis in Model 1, Model 2 (100%), and Model 2 (50%). In Model 1, all solid snacks except almonds were replaced, while in Model 2, healthy snacks were exempted, and 100% or 50% of the remaining solid snacks were replaced. Figure 8 shows the average quantity of solid snacks as well as the quantity of composite almond replacing the snacks in Model 1 and Model 2 for all participants and by age groups. As for energy, the quantity of snacks, as well as non-healthy snacks, was higher among the youngest, i.e., 4–8 years and 9–13 years. The quantity of almonds that replaced all solid snacks is around 60 g (equivalent to two serving sizes) for the youngest and around 50 g for the teenagers and older adults. When healthy snacks are exempted, the quantity of almonds is close to that when replacing all solid snacks for 9–13 years (58 g vs. 64 g) and 14–19 years (48 g vs. 54 g). It is halved when only 50% is replaced, corresponding to around one serving size. The quantity of almonds substituted is much lower than the amount of the initial snack because of the high energy density of the composite almond snack (600 kcal/100 g, Table 2).

## 4. Discussion

Between-meal snacks contributed an average of 22.6% of daily energy to the American diet, with higher percentages observed for children, teenagers, and young adults. Consistent with prior reports [35], solid snacks, which accounted for some 15.5% of dietary energy, were important sources of added sugars, saturated fats, and sodium. The present analyses confirmed that the top snack items in the 2017–23 NHANES were cookies, brownies, cakes and pies, ice cream and frozen dairy desserts, chocolate candy, and doughnuts, pastries, and sweet rolls. Also on the list were corn chips, crackers, and potato chips. As shown by the present analyses, healthy snacks (whole fruits, whole grains, vegetables, yogurts, and nuts) contributed only a small proportion of snack calories.

Compared to typical starchy snacks, almonds contain less carbohydrate and added sugar, less solid (saturated) fats, more protein, fiber, magnesium, and more mono- and polyunsaturated fats (MUFAs and PUFAs). The present substitution analyses that replaced solid snacks with small amounts of almonds led to more nutrient-rich diets that were lower in added sugars, saturated fat, and sodium. Based on the present analyses, replacing snacks with almonds led to diets with higher HEI-2020 scores, reflecting better adherence to the DGA.

For the same number of calories, the modeled dietary patterns had a higher total fat content, as might be expected after replacing sweet bakery goods with nuts. As expected, the fatty acid profile was more favorable, characterized by more MUFAs and PUFAs and more plant omega-3s (alpha-linolenic acid). It is worth noting that almonds were not a vector for sodium. The sodium content in modeled diets was much lower than in observed diets. Given that a small increase was observed for potassium, there was a decline in the dietary sodium to potassium ratio, another index of diet quality. Comparable patterns were observed for almonds-only modeling. These modeled data counter the perceptions that tree nut snacks may act as a vehicle for increasing dietary sodium.

Substitution modeling offers a compelling way to test the nutritional impact of dietary guidance [9,36]. The modeled food patterns can be used to evaluate, compare, and rank the impact of following some very specific dietary advice at the population level. For example, dietary guidelines in the US recommend consuming about 30 g of nuts and seeds per day [6]. Results of modeling studies can quantify the impact of dietary advice and also be used to identify populations that may benefit most from such efforts. For example, we observed that the amounts of nuts necessary to achieve significant improvements in diet quality were relatively small. Adding about 30 g of almonds per day to the regular diet led to improvements in diet quality. What is more, the most significant improvements were obtained for children and adolescents, groups that derive the most energy from snacks and are less likely to select more healthful snacks as compared to older adults. In addition, children and adolescents are less likely to consume tree nuts both overall and as a snack.

The present procedures followed earlier studies [9,36]. In the 2005–2010 NHANES data, usual diets of tree nut consumers were also determined using two 24 h dietary recalls and the NCI Method [37]. Diets of tree nut consumers showed more favorable intakes for vitamins A and C, folate, calcium, iron, magnesium, and zinc, as well as potassium and fiber. The Healthy Eating Index 2005 score (HEI-2005) was higher for tree nut consumers as compared to non-consumers. A later study used substitution modeling [9] and HEI 2010. The composite almond snack developed for this study was weighted by the relative frequency of consumption of different types of almonds. The main form of almonds was unsalted almonds, and they were the most heavily weighted component. Model 1 replaced all solid snacks, whereas Model 2 exempted snacks generally considered to be healthy.

Based on the DGA, making small shifts in eating patterns can make a big difference in diet quality overall [6]. We conducted additional analyses to show that the amounts of almonds were relatively small—replacement and additional analyses pointed to the benefits that could be achieved with only one serving of almonds. Despite major improvements, mean population intakes for fiber, potassium, and sodium remained below recommended values. Strategies to improve diet quality could pair almonds with whole fruit or whole grains to bring more benefits.

Past analyses of NHANES data have linked tree nut consumption with better nutrient adequacy and higher quality diets [38,39]. Prospective cohort studies have also observed that the consumption of tree nuts reduces the risk of all-cause mortality, deaths due to heart disease, cancer, and respiratory disease, and the incidence of heart disease and may reduce the likelihood of weight gain [14,15,16,17,18,19]. Almonds have been featured in the DGA and in numerous reports by professional and advocacy groups, including the American Heart Association, the American Diabetes Association, and the Academy of Nutrition and Dietetics [40].

Based on USDA reports, the consumption of tree nuts, whole grains, plant and seafood protein, and whole fruits and vegetables lags well below recommended levels [41]. However, this may be changing. The consumption of almonds, pistachios, and other tree nuts is growing in the US and globally [42]. Analyses of NHANES dietary trends suggest that the consumption of tree nuts rose from 0.11 to 0.27 oz equivalents per day from 1999–2000 to 2011–2012 [43].

The present study has a number of strengths. First, the NHANES data are representative of the US population. Second, the impact of replacement modeling on diet quality was tested using multiple diet quality metrics. Third, we used a composite almond snack that matched population eating habits. Therefore, the modeled results are representative of what would be observed if current consumption patterns continued.

The study also has limitations. NHANES data were based on self-reports. Data for young children were obtained by proxy reports from parents or caregivers. Not all nuts were clearly identified; it may be that some NHANES participants reported tree nuts while eating peanuts [44]. Body mass indices of NHANES participants were not considered. Models also have limitations. Substitution models may provide different results when the replacement is per calorie or per serving. Given the diversity of snack foods and a wide range of serving sizes, we opted for an isocaloric substitution model. Although such a model would (theoretically) not increase energy intakes, the higher satiating value of almonds might bring about a reduction in energy intakes [45,46]. Studies have noted that adding nuts [47], and specifically almonds [48], to a regular diet does not lead to an increase in body weight. Although the cross-sectional nature of NHANES does not allow for establishing causality, improvements in diet quality ought to lead to benefits to health. Diet quality can be improved by making some small changes in food choices.

The present analyses suggest that the most significant improvements in diet quality were obtained for children, teenagers, and young adults. Those are the groups that derive most energy from snacks. The USDA Smart Snacks Standards [49] state that almonds, dry roasted without salt in 1 oz packages (172 kcal) [50] provide <200 kcal per serving and comply with the school guidelines. Dried fruit with nuts and/or seeds with no added sugars or fats also comply with the guidelines. Tree nuts generate satiety, and their consumption does not appear to lead to excess weight gain [47,48]. Economic initiatives might address the cost of nuts relative to less healthful snacks in order to increase consumption.

## 5. Conclusions

Based on present modeling analyses, widespread replacement of solid food snacks with tree nuts or almonds would improve the quality of the diet. Even a partial replacement, exempting already nutrient-rich snacks, had a significant positive effect on diet quality. Substitution modeling can inform implementation of the 2025–30 DGA.

## Figures and Tables

**Table 1:** Total calories and calories by snack type by NHANES sociodemographic variables.

	N	Total Diet (kcal)		Snacks					
				Total Snacks (kcal)		Less Healthy Snacks (kcal)		Healthy Snacks (kcal)	
		Mean	SE	Mean	SE	Mean	SE	Mean	SE
All	15,258	2019	17.6	328.8	2.60	270.5	2.35	58.3	1.00
Gender									
Female	8071	1766	14.6	304.9	3.10	251.3	2.81	53.6	1.18
Male	7187	2286	18.0	354.0	4.23	290.6	3.83	63.3	1.64
*p*		<0.001		<0.001		<0.001		0.003	
Age group									
4–8 y	1343	1715	12.6	363.0	7.08	309.5	6.48	53.5	2.61
9–13 y	1425	1947	14.4	390.4	8.54	354.0	7.95	36.3	1.77
14–19 y	1565	1950	18.9	327.1	8.39	291.4	7.88	35.7	2.35
20–30 y	1500	2096	18.1	302.8	9.00	260.0	8.41	42.8	2.67
31–50 y	3171	2137	18.6	323.4	5.71	261.5	5.11	61.9	2.17
51–70 y	4372	2033	17.5	332.6	4.84	257.8	4.24	74.8	2.30
>70 y	1882	1853	15.0	307.5	6.74	242.3	5.94	65.2	3.01
*p*		<0.001		<0.001		<0.001		<0.001	
Ethnicity									
Mexican	1642	2011	18.1	292.0	7.07	235.6	6.53	56.4	2.43
NH Black	3399	1939	19.3	319.9	6.14	276.2	5.81	43.8	1.76
NH White	6549	2054	16.9	344.3	3.95	282.6	3.49	61.6	1.67
Other H	1496	1922	19.8	292.3	8.82	244.5	8.35	47.9	2.34
Other	2172	2000	17.2	315.0	6.21	248.8	5.67	66.2	2.53
*p*		<0.001		0.001		0.001		<0.001	
IPR *									
<1	2735	1956	20.7	304.3	6.95	267.7	6.56	36.6	1.84
1–1.99	3257	1957	18.4	304.6	5.41	254.5	4.93	50.1	1.96
2–3.49	3039	2064	17.4	348.3	5.80	287.0	5.28	61.3	2.10
≥3.5	4502	2069	16.2	344.4	4.64	275.4	4.09	69.0	2.11
*p*		0.001		0.002		0.118		<0.001	

* IPR = Income-to-poverty ratio.

**Table 2:** Nutrient composition per 100 kcal of all solid snacks and the composite almond snack.

Nutrients	All Solid Snacks		Composite Almond Snack		*p*-Value
	Mean	SE	Mean	SE	
Amount, g	50.5	0.413	16.5	0.000	<0.001
Energy, kcal	100	0.008	100	0.064	<0.001
Carbohydrates, g	13.7	0.041	3.4	0.011	<0.001
Fibers, g	1	0.007	1.7	0.004	<0.001
Proteins, g	2.3	0.014	3.4	0.005	<0.001
Total fats, g	4.3	0.016	8.8	0.009	<0.001
Saturated fats, g	1.4	0.008	0.7	0.003	<0.001
Monounsaturated fats, g	1.5	0.007	5.5	0.006	<0.001
Polyunsaturated fats, g	1	0.006	2.2	0.003	<0.001
Magnesium, mg	13	0.070	44.6	0.065	<0.001
Sodium, mg	103	1.187	22.1	0.956	<0.001
Added sugars, g	4.2	0.031	0.1	0.014	<0.001
Oils, g	1.5	0.013	5.8	0.010	<0.001
Solid fats, g	2	0.014	0.0	0.003	<0.001

**Table 3:** Percent of calories from eligible solid snacks by What We Eat in America (WWEIA) food category in Model 1 and Model 2.

WWEIA Category	Model 1	Model 2
Ice cream and frozen dairy desserts	9.6	11.5
Cookies and brownies	9.2	11.1
Cakes and pies	7.4	8.9
Candy containing chocolate	4.4	5.3
Tortilla, corn, other chips	4.3	5.2
Doughnuts, sweet rolls, pastries	3.5	4.2
Potato chips	3.4	4.1
Crackers, excludes saltines	3.4	4.1
Popcorn	3.4	4.1
Candy not containing chocolate	2.7	3.3
Cheese	2.7	3.2
Pretzels/snack mix	2.0	2.4
Cereal bars	1.6	1.9
Biscuits, muffins, quick breads	1.5	1.8
Pizza	1.3	1.6
RTE cereal, higher sugar (>21.2 g/100 g)	1.1	0.7
Yeast breads	1.1	1
Dips, gravies, other sauces	1	1.2
Milkshakes and other dairy drinks	1	1.2
Others	24.8	23.4
**Not replaced in Model 2**		
Nuts and seeds	7.1	-
Bananas	1.9	-
Apples	1.8	-

**Table 4:** HEI-2020 values for observed, model 1, and model 2 (100 and 50%).

		N	Observed		Model 1			Model 2_50			Model 2_100		
			Mean	SE	Mean	SE	*p*-Value ^1^	Mean	SE	*p*-Value ^1^	Mean	SE	*p*-Value ^1^
**All**		15,258	52.4	0.11	59.6	0.10	<0.001	57.8	0.10	<0.001	60.6	0.10	<0.001
**Sex**	Female	8071	53.4	0.15	60.9	0.14	<0.001	59.0	0.14	<0.001	62.0	0.14	<0.001
	Male	7187	51.2	0.16	58.3	0.14	<0.001	56.4	0.15	<0.001	59.2	0.15	<0.001
	*p*-value		<0.001		<0.001			<0.001			<0.001		
**Age group**	4–8 y	1343	52.8	0.32	64.0	0.30	<0.001	61.4	0.31	<0.001	65.6	0.31	<0.001
	9–13 y	1425	48.2	0.32	59.9	0.30	<0.001	56.7	0.31	<0.001	61.1	0.31	<0.001
	14–19 y	1565	47.2	0.32	56.1	0.31	<0.001	53.7	0.31	<0.001	57.0	0.32	<0.001
	20–30 y	1500	49.4	0.34	55.8	0.31	<0.001	54.1	0.32	<0.001	56.7	0.32	<0.001
	31–50 y	3171	52.3	0.24	58.6	0.22	<0.001	57.0	0.23	<0.001	59.6	0.23	<0.001
	51–70 y	4372	55.1	0.20	61.2	0.18	<0.001	59.7	0.19	<0.001	62.2	0.18	<0.001
	>70 y	1882	56.7	0.32	63.5	0.28	<0.001	61.8	0.29	<0.001	64.4	0.29	<0.001
	*p*-value		<0.001		<0.001			<0.001			<0.001		
**Race/Ethnicity**	Mexican	1642	51.9	0.30	58.3	0.28	<0.001	56.9	0.29	<0.001	59.6	0.29	<0.001
	Non-Hispanic Black	3399	50.9	0.22	57.9	0.21	<0.001	56.1	0.21	<0.001	58.6	0.21	<0.001
	Non-Hispanic White	6549	52.2	0.17	59.9	0.15	<0.001	57.8	0.16	<0.001	60.8	0.16	<0.001
	Other Hispanic	1496	53.5	0.34	59.9	0.32	<0.001	58.5	0.33	<0.001	61.0	0.33	<0.001
	Other race	2172	54.3	0.31	60.9	0.27	<0.001	59.5	0.28	<0.001	62.3	0.28	<0.001
	*p*-value		<0.001		<0.001			<0.001			<0.001		
**IPR** *	<1	2735	49.1	0.25	56.6	0.24	<0.001	54.6	0.24	<0.001	57.5	0.25	<0.001
	1–1.99	3257	50.4	0.23	57.8	0.22	<0.001	55.9	0.22	<0.001	58.7	0.22	<0.001
	2–3.49	3039	51.3	0.24	58.9	0.22	<0.001	57.1	0.23	<0.001	60.1	0.23	<0.001
	≥3.5	4502	54.7	0.20	61.8	0.18	<0.001	59.9	0.19	<0.001	62.7	0.18	<0.001
	*p*-value		<0.001		<0.001			<0.001			<0.001		

* IPR = Income-to-poverty ratio, ^1^ *t*-test with observed value.

**Table 5:** Nutrient composition of observed and modeled diets for all NHANES participants. Model 1 and Model 2 (50% and 100%).

Total NHANES Sample	Observed		Model 1			Model 2_50			Model 2_100		
Nutrient/Score	Mean	SE	Mean	SE	*p*-Value ^1^	Mean	SE	*p*-Value ^1^	Mean	SE	*p*-Value ^1^
Amount, g	3120	11.81	3030	11.69	<0.001	3090	11.77	<0.001	3070	11.75	<0.001
Energy, kcal	2019	6.40	2019	6.40	0.165	2019	6.40	0.165	2019	6.40	0.165
Carbohydrates, g	234	0.82	203	0.72	<0.001	222	0.77	<0.001	209	0.73	<0.001
Total fats, g	83.4	0.31	97.3	0.37	<0.001	89	0.33	<0.001	94.6	0.36	<0.001
Monounsaturated fats, g	28.3	0.11	41	0.18	<0.001	33.6	0.14	<0.001	38.9	0.17	<0.001
Polyunsaturated fats, g	19.4	0.09	23.2	0.10	<0.001	21	0.09	<0.001	22.6	0.10	<0.001
Oils, g	28.6	0.15	42.4	0.20	<0.001	34.5	0.17	<0.001	40.4	0.20	<0.001
Solid fats, g	35.1	0.17	28	0.14	<0.001	31.6	0.15	<0.001	28.1	0.14	<0.001
Fibers, % DRI	61.1	0.27	71.9	0.30	<0.001	67	0.28	<0.001	72.9	0.30	<0.001
Proteins, % DRI	166	0.62	174	0.65	<0.001	169	0.63	<0.001	173	0.64	<0.001
Saturated fats, % DRI	120	0.27	109	0.24	<0.001	114	0.25	<0.001	108	0.24	<0.001
Magnesium, % DRI	86.4	0.34	121	0.56	<0.001	102	0.42	<0.001	117	0.53	<0.001
Sodium, % DRI	167	0.59	153	0.55	<0.001	160	0.57	<0.001	153	0.55	<0.001
Added sugars, % DRI	124	0.66	98.7	0.63	<0.001	112	0.63	<0.001	99.6	0.63	<0.001
NRF	455	1.28	509	1.18	<0.001	488	1.22	<0.001	514	1.20	<0.001
MAR	53.5	0.07	56.6	0.08	<0.001	55.7	0.08	<0.001	56.9	0.08	<0.001
MER	30.8	0.17	22.8	0.15	<0.001	26.4	0.16	<0.001	23	0.15	<0.001

^1^ *t*-Test with observed value.

**Table 6:** Diet quality metrics for observed diets and modeled food patterns (Model 3). Shown are values for NRF9.3, MAR, MER, and HEI-2020. Data are for entire sample, n = 15,258.

Nutrient Density Metric	Observed		Model 3 (30 g)			Model 3 (50 g)		
	Mean	SE	Mean	SE	*p*-Value ^1^	Mean	SE	*p*-Value ^1^
Amount, g	3120	11.81	3150	11.81	<0.001	3170	11.81	<0.001
Energy, kcal	2019	6.40	2201	6.40	<0.001	2322	6.40	<0.001
NRF9.3	455	1.28	488	1.17	<0.001	505	1.11	<0.001
MAR	53.5	0.07	59.9	0.07	<0.001	63.3	0.06	<0.001
MER	30.8	0.17	25.3	0.16	<0.001	22.8	0.15	<0.001
HEI-2020	52.4	0.11	59.2	0.10	<0.001	61.4	0.09	<0.001

^1^ *t*-Test with observed value.

## Data Availability

The datasets supporting the conclusions of this article are available in a public repository as described below. The authors do not own the data. The NHANES data are available from the National Center for Health Statistics website: https://wwwn.cdc.gov/nchs/nhanes/analyticguidelines.aspx (accessed on 9 October 2025). The FPED is available from the United States Department of Agriculture: http://ars.usda.gov/northeast-area/beltsville-md-bhnrc/beltsville-human-nutrition-research-center/food-surveys-research-group/docs/fped-databases/ (accessed on 9 October 2025). The FNDDS is available from the United States Department of Agriculture: https://www.ars.usda.gov/northeast-area/beltsville-md-bhnrc/beltsville-human-nutrition-research-center/food-surveys-research-group/docs/fndds/ (accessed on 9 October 2024).

## Supplementary Materials

The following supporting information can be downloaded at: https://www.mdpi.com/article/10.3390/nu18010087/s1, Supplemental Table S1. Summary of reference nutrient values from the Dietary Reference Intake (DRI) found in the DGA 2020-2025 and those issued by the Institute of Medicine.
