# Supplementary material for: Replacing Solid Snacks with Almonds or Adding Almonds to the Diet Improves Diet Quality and Compliance with the 2020–25 Dietary Guidelines for Americans: Modeling Analyses of NHANES 2017–23 Data
Source: Nutrients. 2025 Dec 26;18(1):87. doi: 10.3390/nu18010087 (PMC12787779; doi:10.3390/nu18010087)
Supplement: Supplementary file 1 [file nutrients-18-00087-s001.zip › nutrients-4033143-supplementary.pdf]

Replacing Solid Snacks with Almonds or Adding Almonds to the Diet Improves Diet Quality and Compliance with the 2020–25 Dietary Guidelines for Americans: Modeling Analyses of NHANES 2017–23 Data

Mattieu Maillot [1], Romane Poinsot [1], Maha Tahiri [2] and Adam Drewnowski [3,*]

Table S1. Summary of reference nutrient values from the Dietary Reference Intake (DRI) found in the DGA 2020-2025 and those issued by the Institute of Medicine.

Nutrient	Sex	4-8	9-13	14-18	19-30	31-50	51-70	71-100
Proteins (g/d)	Male	19	34	52	56	56	56	56
	Female	19	34	46	46	46	46	46
Fibers (g/d)	Male	20	25	31	34	31	28	28
	Female	17	22	25	28	25	22	22
LA* (g/d)	Male	10	12	16	17	17	14	14
	Female	10	10	11	12	12	11	11
ALA** (g/d)	Male	0.9	1.2	1.6	1.6	1.6	1.6	1.6
	Female	0.9	1	1.1	1.1	1.1	1.1	1.1
Calcium (mg/d)	Male	1000	1300	1300	1000	1000	1000	1200
	Female	1000	1300	1300	1000	1000	1200	1200
Iron (mg/d)	Male	10	8	11	8	8	8	8
	Female	10	8	15	18	18	8	8
Magnesium (mg/d)	Male	130	240	410	400	420	420	420
	Female	130	240	360	310	320	320	320
Potassium (mg/j)	Male	2300	2500	3000	3400	3400	3400	3400
	Female	2300	2300	2300	2600	2600	2600	2600
Zinc (mg/d)	Male	5	8	11	11	11	11	11
	Female	5	8	9	8	8	8	8
Copper (mg :d)	Male	0.44	0.7	0.89	0.9	0.9	0.9	0.9
	Female	0.44	0.7	0.89	0.9	0.9	0.9	0.9
Selenium (µg/d)	Male	30	40	55	55	55	55	55
	Female	30	40	55	55	55	55	55
Vitamin A (ER eq/d)	Male	400	600	900	900	900	900	900
	Female	400	600	700	700	700	700	700
Vitamin E (mg/d)	Male	7	11	15	15	15	15	15
	Female	7	11	15	15	15	15	15
Vitamin D (µg/d)	Male	15	15	15	15	15	15	20
	Female	15	15	15	15	15	15	20

Vitamin C (mg/d)	Male	25	45	75	90	90	90	90
	Female	25	45	65	75	75	75	75
Vitamin B1 (mg/d)	Male	0.6	0.9	1.2	1.2	1.2	1.2	1.2
	Female	0.6	0.9	1	1.1	1.1	1.1	1.1
Vitamin B2 (mg/d)	Male	0.6	0.9	1.3	1.3	1.3	1.3	1.3
	Female	0.6	0.9	1	1.1	1.1	1.1	1.1
Vitamin B3 (mg/d)	Male	8	12	16	16	16	16	16
	Female	8	12	14	14	14	14	14
Vitamin B6 (mg/d)	Male	0.6	1	1.3	1.3	1.3	1.7	1.7
	Female	0.6	1	1.2	1.3	1.3	1.5	1.5
Vitamin B12 (µg/d)	Male	1.2	1.8	2.4	2.4	2.4	2.4	2.4
	Female	1.2	1.8	2.4	2.4	2.4	2.4	2.4
Vitamin B9 (µg/j)	Male	200	300	400	400	400	400	400
	Female	200	300	400	400	400	400	400
SFA*** (%EI)	Male	10	10	10	10	10	10	10
	Female	10	10	10	10	10	10	10
Added sugars (% EI)	Male	10	10	10	10	10	10	10
	Female	10	10	10	10	10	10	10
Sodium (mg/d)	Male	1500	1800	2300	2300	2300	2300	2300
	Female	1500	1800	2300	2300	2300	1200	1200

*LA: Linoleic Acide; **ALA: alpha-linolenic acid; ***SFA: Saturated Fatty Acids
